# An Electromagnetic-Driven Microshutter Array in a Field-of-View Gated Image System for All-Time Star Sensors

**DOI:** 10.3390/mi14020452

**Published:** 2023-02-15

**Authors:** Liang Fang, Weimin Wang, Qiang Wang, Shuliang Tan, Hui Zhang, Rujin Zhao, Enhai Liu

**Affiliations:** 1 Institute of Optics and Electronics, Chinese Academy of Sciences, Chengdu 610209, China; 2 Key Laboratory of Science and Technology on Space Optoelectronic Precision Measurement, Chinese Academy of Sciences, Chengdu 610209, China; 3 University of Chinese Academy of Sciences, Beijing 100049, China; 4 Defense Key Disciplines Laboratory of Novel Micro-Nano Devices and System Technology, Chongqing University, Chongqing 400044, China

**Keywords:** microshutter array, electromagnetic actuation, field of view gated

## Abstract

Aiming at the application requirements of a field of view (FOV) gated imaging system for all-time star sensors, a key device of a microshutter array with large unit size, high duty cycle, and fast response speed based on the electromagnetic actuation is designed. The proposed microshutter array adopts the principle that the current-carrying coil is subjected to the magnetic force in the magnetic field. The coil element is deflected by the loading current and acts as a light barrier in realizing the optical switch function. The effects of the coil element parameters on the magnetic force torque, torsion beam resistance torque, and switch response time are analyzed, and the structural parameters of the coil element are determined. A sample of the proposed microshutter array based on the electromagnetic actuation with a 4-mm period and a 2.8-mm aperture is fabricated and tested. The test results demonstrate the good switching function of the proposed microshutter array and show that the switch response time of the microshutter element is approximately 2.5 ms. This proposed microshutter array is used to gate an instantaneous small FOV to suppress the sky’s background radiation and make a FOV-gated imaging system realize the multi-stars detection by switching the gated FOV rapidly. This will solve the problem that only one star can be detected within the FOV by a traditional all-time star tracker and promote the all-time star sensor to realize star pattern recognition and autonomous astronomical navigation in the daytime.

## 1. Introduction

A star sensor is a type of space attitude measuring device based on stars, which has the advantages of high precision, strong anti-interference, and autonomous attitude determination. It has thus been widely used in satellite platforms [1,2,3,4]. For near-earth space platforms inside the atmosphere, such as ships, airplanes, and balloons, satellite navigation systems are widely used. However, satellite navigation systems are vulnerable to hostile jamming and reliability failures [5]. By extending a star sensor from outer space to near- earth space, the problem of high dependence on a satellite navigation system for near-earth space platforms can be solved, which has a wide application prospect [6,7,8]. Compared with a space-based star sensor, a near- earth space star sensor faces the disturbance of a strong sky background light. Therefore, to detect weak star targets in a strong sky background, it is necessary to use methods for suppressing the sky background light and improving the detection signal-to-noise ratio of a system. At present, the most common method for sky background light suppression has been to filter the spectrum and reduce the solid angle of a single pixel of a detector [9,10,11,12]. The daytime star detection systems are usually designed with a small field of view (FOV), and equipped with a servo mechanism (e.g., mechanical turntable) to track a single star in the FOV. The servo mechanism increases the system’s volume and weight [13]. In 2008, Trex Enterprises proposed a daytime stellar imager consisting of three telescopes with a large aperture and narrow FOV operating at SWIR waveband [5]. Although the servo mechanism was not used in this scheme, it was difficult to meet the application requirements of airborne platforms due to the large volume and weight caused by the three sets of telescopes with a large aperture.

The optical imaging system based on the FOV-gated technology [14] adopts a large-FOV telescope to collect information on stars and uses microlens and microshutter array to gate the instantaneous FOV rapidly. This can simultaneously ensure a large FOV and a strong sky background light suppression effect. This system has the capability of realizing multi-star detection and star pattern recognition during the daytime and thus is particularly suitable for the application to all-time star sensors. However, the FOV-gated optical imaging system requires using a microshutter array to realize the function of switching the gated FOV quickly, requiring the microshutter array to have the characteristics of large unit size, high duty cycle, and high response speed.

A microshutter array based on electrostatic actuation is developed by the near-infrared spectrometer research team of James Webb Space Telescope (JWST) [15,16,17]. The developed microshutter array has a pixel pitch of 100 µm × 200 µm, a shutter blade size of 87 µm × 186 µm, and several microshutter elements of 365 × 171. It used to select multiple astronomical targets to enable their spectra to be simultaneously measured. Each microshutter can be commanded to open or close to match the position of targets in any astronomical field. The team’s newly developed NexGen Micro-Shutter Array (NGMSA) technology [18] intends to achieve a larger array format in order to meet the requirements of larger FOV for future telescopes in space. A similar microshutter array based on electrostatic actuation is developed for a ground-based infrared telescope at the Tokyo Atacama Observatory on a 5640-m-high mountain in Chile [19]. A trial sub-cluster microshutter array with 40 × 10 elements has been fabricated in a 20 mm × 20 mm area, with a shutter blade size of 100 μm × 1000 μm. Comparatively, the element size of the microshutter required in a FOV-gated optical imaging system is larger. Because each microshutter element must be combined with a milimeter-aperture microlens element to obtain FOV-gated near diffraction limit imaging, the element size of the microshutter is required to reach the millimeter level. Therefore, if the electrostatic driving scheme is adopted, it will be challenging to control the stress of each microshutter element with a large element size in actual processing.

Another option for a microshutter array is a roll-up mechanism based on electrothermal actuation. A unique electrothermal bimorph cantilever plate can be designed to bend 90° at rest and stay flat at low voltage by using the difference of thermal expansion coefficients of the layered materials. A microshutter array with an element size of 700 μm × 800 μm and a response time of about 15 ms can be fabricated for smart windows [20]. It is still difficult to obtain the large element size due to the challenge of the stress control.

Electromagnetic actuation is also a common driving method [21,22]. However, because the size of an electromagnetic actuator is generally large, due to the required coil components, it is often used for a single camera shutter. There is no published research regarding microshutter arrays based on electromagnetic actuation. In this paper, an electromagnetic-driven microshutter array based on flexible circuit board processing technology is proposed to solve the problem that it is difficult to fabricate microshutter arrays with large element size by the traditional bulk silicon micromachining technology. By designing and fabricating the microshutter element directly on the flexible circuit board, the stress control problem in the bulk silicon micromachining is avoided, which is suitable for machining microshutter with a large element size required in the FOV-gated optical imaging system. In addition, the proposed microshutter array has the advantages of high duty ratio and fast response speed. It can turn a FOV-gated imaging system into a practical application and enable all-time star sensors to perform multi-star detection, star pattern recognition, and autonomous astronomical navigation during the daytime.

## 2. Design Requirements of a Microshutter Array in FOV-Gated Imaging System

**Figure 1:**
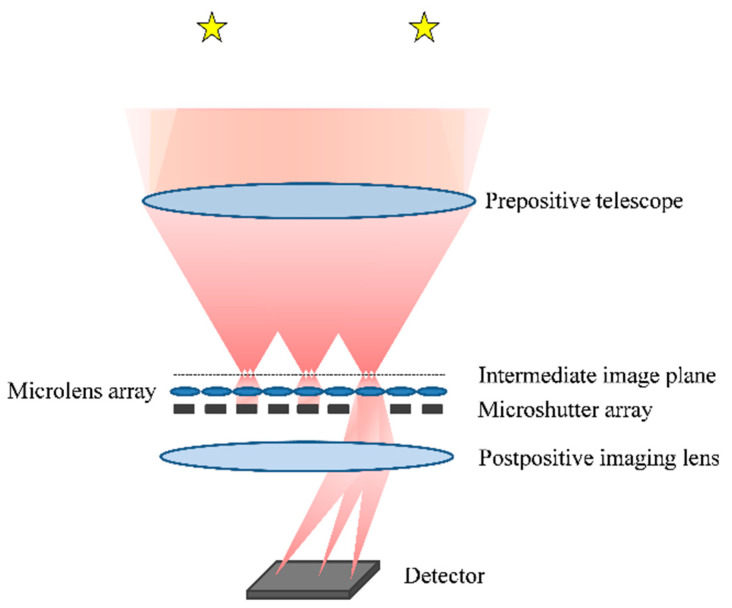
Schematic diagram of a FOV-gated imaging system for all-time star sensors.

The optical path schematic diagram of a FOV-gated imaging system for all-time star sensors is presented in Figure 1. As shown in Figure 1, the system consists of three main components: a prepositive telescope with a telecentric beam path in the image space, a microlens and microshutter array, and a common postpositive imaging lens. Multiple stars are imaged on the intermediate image plane by the prepositive telescope with a large FOV, and the large FOV on the intermediate image plane is subdivided and gated by the microshutter and microlens arrays. The microlens element is combined with the postpositive imaging lens to magnify the stellar image on the intermediate image plane. The purpose of magnification imaging is to increase the focal length of an imaging channel, reducing the single-pixel FOV and suppressing the atmospheric background radiation. Since each microlens element has its own optical axis, which is parallel to the optical axis of the postpositive imaging lens, there are many imaging channels in the system. Through the quick switching of a microshutter, the stellar images in different gated FOV can be realized in a common detector. The FOV-gated optical imaging system combines the advantages of a wide FOV telescope and narrow gated FOV imaging, thus achieving a strong sky background radiation suppression effect within a wide FOV. This system is expected to realize multi-star detection and star pattern matching during the daytime.

**Figure 2:**
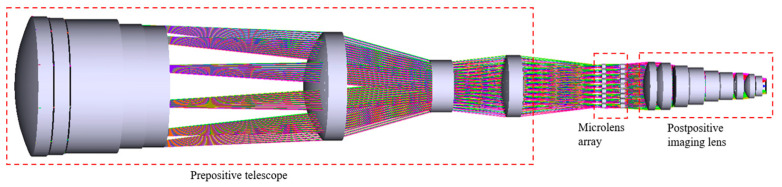
The designed optical path diagram of a FOV-gated optical imaging system.

To verify the FOV-gated imaging function, a FOV-gated imaging system working in the short-wave infrared band was designed [14], as shown in Figure 2. The working band of this system is 1.3 µm–1.7 µm, the aperture is Φ100 mm, the FOV of the prepositive telescope is 6°, and the total focal length is 1.47 m; the instantaneous gated FOV is 0.4°, the aperture of the microlens element is 3.5 mm, the pixel size of the detector is 20 μm × 20 μm, the corresponding single-pixel FOV is 2.8″, and the pixel number is 640 × 512. This system can realize near-diffraction limit imaging in a gated FOV, and the energy concentration of a stellar image within 3 × 3 pixels is above 80%.

**Figure 3:**
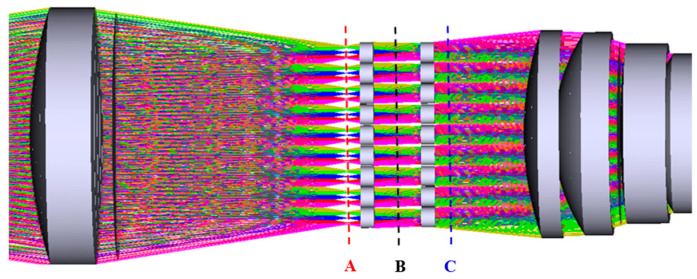
An enlarged view of the area near the microlens presented in Figure 2. Dashed lines A, B, and C represent three optional positions for placing a microshutter array.

The microshutter array is a key device of the optical imaging system allowing the systemto switch the gated FOV rapidly, and its period is the same as that of the microlens array. The distribution of microshutter elements has a one-to-one correspondence with that of the microlens elements. An enlarged view of the area near the microlens in Figure 2 is displayed in Figure 3. As shown in Figure 3, there are three optional positions in the system for placing a microshutter array to realize the gating of a narrow instantaneous FOV: the intermediate image plane of the system, the middle of the microlens array group, and the rear of the microlens array group, which are denoted in Figure 3 by A, B, and C, respectively.

If a microshutter array is placed in position B or C, the clear aperture of a microshutter element will be almost the same as that of a microlens. However, if a microshutter array is placed in position A, the clear aperture of a microshutter element will be smaller than that of a microlens. Therefore, when the period of a microlens array is constant, placing a microshutter array in position A can reduce the requirement for a duty cycle of a microshutter array, which is beneficial to decrease the design and processing complexity of a microshutter array.

**Figure 4:**
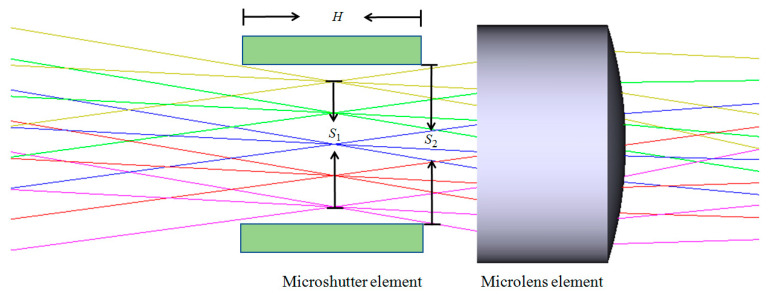
The light path diagram of a microshutter element placed on the intermediate image plane. Lines in different colors present light rays in different FOV.

The light path diagram of a microshutter element placed on the intermediate image plane is presented in Figure 4. As the focal length of the prepositive telescope in the proposed system is 300 mm, the size of a gated FOV is 0.4°, and the corresponding size of the intermediate image plane $S_{1}=300\;\mathrm{mm}\times \tan(0.4{}^{\circ})\approx 2.1\;\mathrm{mm}$. Considering that the microshutter element has a certain thickness, and the light beam has a divergence angle when passing through the microshutter element, the actual clear aperture of the microshutter element should be larger than 2.1 mm. Assume that the thickness of the microshutter element is *H* = 4 mm and the intermediate image plane is located in the middle of the microshutter element. Then, since the relative aperture of the prepositive telescope is 1/3, the clear aperture of the microshutter element should be at least $S_{2}=S_{1}+\frac{1}{3}\cdot \frac{H}{2}\approx 2.8\;\mathrm{mm}$. Further, to ensure the side length duty cycle of the microshutter element is not less than 70%, the period of the microshutter array should be not more than 4 mm. Finally, considering the response time requirement of a microshutter array in practical applications, the main design indexes of the microshutter element are set as shown in Table 1.

## 3. Microshutter Array Design

### 3.1. Microshutter Array Based on Electromagnetic Actuation

**Figure 5:**
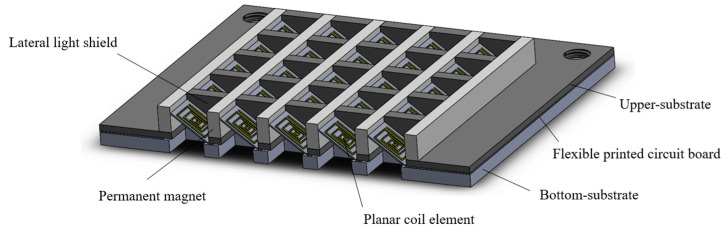
Schematic diagram of a microshutter array based on the electromagnetic actuation.

The schematic diagram of a microshutter array based on the electromagnetic actuation is presented in Figure 5. The microshutter array consists of a permanent magnet array, a lateral light shield array, an upper-substrate, a flexible printed circuit board (FPCB) with a planar coil array, and a bottom-substrate. The lateral light shield array is fabricated on the upper-substrate and combined with the permanent magnet array to form a rectangular grid. A planar coil array can be fabricated in a FPCB, and the FPCB bonded to the bottom-substrate. Each planar coil element is placed between two permanent magnets, leaning on one of them and acting as a microshutter element. The electrode of each planar coil is extracted by a FPCB and used as a torsion beam of the planar coil. It should be noted that when a planar coil element carries the current, it will deflect with the torsion beam as the axis by the electromagnetic force in the magnetic field, so the microshutter element is turned on. When the current in the planar coil element is removed, the planar coil element will deflect in the reverse direction by the restoring force of the torsion beam, closing the microshuter element. There is a square hole array fabricated on the bottom-substrate, where each square hole corresponds to a microshutter element and the side length of a square hole element determines the clear aperture of a microshutter element. The microshutter array can meet the application requirements for millimeter-level cell size, high duty cycle, high response speed, and low cost without using complex MEMS processing technology.

### 3.2. Electromagnetic Coil Element Design

There are three main factors that affect the deflection of a planar coil element: the torque of electromagnetic force, the gravity torque, and the resistance torque of the torsion beam. Because the planar coil element is fabricated in a FPCB, its thickness is extremely thin, so the gravity torque can be neglected. The torque of the electromagnetic force and the resistance torque of the torsion beam are closely related to the structural parameters of a planar coil element. In addition, the structural parameters of a planar coil element affect the response time of a microshutter. Therefore, the influence of coil structure parameters on the electromagnetic force torque, torsion beam resistance torque, and switch response time are analyzed below.

#### 3.2.1. Magnetic Field Distribution in Microshutter Element

The electromagnetic force received by a coil element is closely related to the magnetic field distribution, so it is necessary to determine the magnetic field distribution of the two rectangular permanent magnets in a microshutter element.

**Figure 6:**
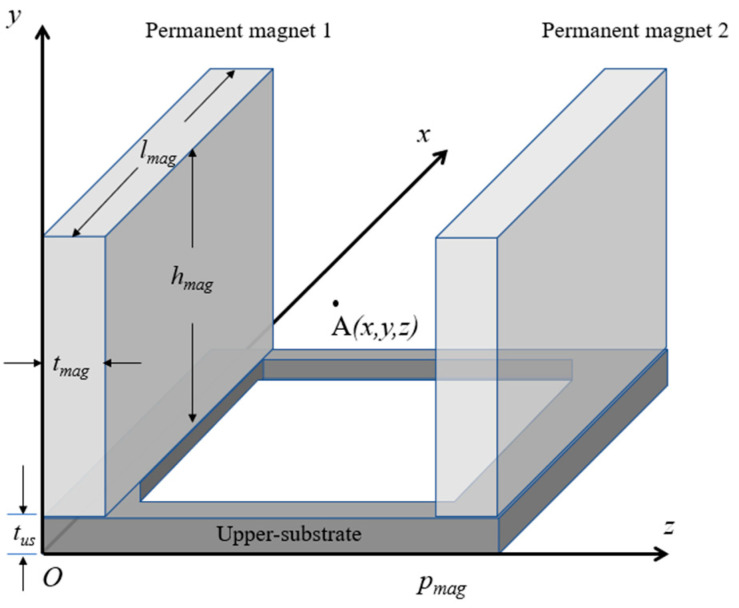
The rectangular coordinate system established for calculating the magnetic field distribution of rectangular permanent magnets.

Assume that the length, height, and thickness of each permanent magnet in a microshutter element are denoted by *l_mag_*, *h_mag_*, and *t_mag_*, respectively. Further, establish a rectangular Cartesian coordinate system with the lower left corner of the upper -substrate in a microshutter element as the coordinate origin, as shown in Figure 6. Then, the magnetic field intensity generated by permanent magnet 1 at point A(*x*, *y*, *z*) in the space between two permanent magnets is given by [23]:

(1) $$B_{1x}=\int_{0}^{t_{mag}}dB_{1x}=-\frac{K}{2}[\Gamma \left(l_{mag}-x,y-t_{us},z\right)+\Gamma \left(l_{mag}-x,h_{mag}+t_{us}-y,z\right)-\;\Gamma \left(x,y-t_{us},z\right)-\Gamma \left(x,h_{mag}+t_{us}-y,z\right)]{|}_{0}^{t_{mag}},$$

(2) $$B_{1y}=\int_{0}^{t_{mag}}dB_{1y}=-\frac{K}{2}[\Gamma \left(h_{mag}+t_{us}-y,x,z\right)+\Gamma \left(h_{mag}+t_{us}-y,l_{mag}-x,z\right)-\;\;\Gamma \left(y-t_{us},x,z\right)-\Gamma \left(y-t_{us},l_{mag}-x,z\right)]{|}_{0}^{t_{mag}},$$

(3) $$B_{1z}=\int_{0}^{t_{mag}}dB_{1z}=-K[\varphi \left(y-t_{us},l_{mag}-x,z\right)+\varphi \left(h_{mag}+t_{us}-y,l_{mag}-x,z\right)+\;\;\varphi \left(x,h_{mag}+t_{us}-y,z\right)+\varphi \left(l_{mag}-x,h_{mag}+t_{us}-y,z\right)+\varphi \left(h_{mag}+t_{us}-y,x,z\right)\;+\varphi \left(y-t_{us},x,z\right)+\varphi \left(l_{mag}-x,y-t_{us},z\right)+\varphi \left(x,y-t_{us},z\right)]{|}_{0}^{t_{mag}},$$

where $K=\frac{\mu_{0}J}{4\pi }$, where $\mu_{0}$ is permeability of vacuum, and $\mu_{0}=4\pi \times 10^{-7}$; *J* is the equivalent surface current density of the permanent magnet; Γ is a functional symbol representing independent variables $\left(\alpha_{1},\alpha_{2},\alpha_{3}\right)$; *φ* is a functional symbol indicating independent variables $\left(\beta_{1},\beta_{2},\beta_{3}\right)$; the two functional symbols are expressed as follows:

(4) $$\Gamma \left(\alpha_{1},\alpha_{2},\alpha_{3}\right)=ln\frac{\sqrt{\alpha_{1}^{2}+\alpha_{2}^{2}+{\left(\alpha_{3}-z_{0}\right)}^{2}}-\alpha_{2}}{\sqrt{\alpha_{1}^{2}+\alpha_{2}^{2}+{\left(\alpha_{3}-z_{0}\right)}^{2}}+\alpha_{2}},$$

(5) $$\varphi \left(\beta_{1},\beta_{2},\beta_{3}\right)=\{\begin{array}{cc} arctan[\frac{\beta_{1}\left(\beta_{3}-z_{0}\right)}{\beta_{2}\sqrt{\beta_{1}^{2}+\beta_{2}^{2}+{\left(\beta_{3}-z_{0}\right)}^{2}}}] & \left(\beta_{2}\neq 0\right),\\ \;\;\;\;\;\;\;\;\;0\;\;\;\;\;\;\;\;\;\;\;\;\; & \left(\beta_{2}=0\right). \end{array}$$

In Equations (1)–(3), symbol $\left[\;\right]{|}_{0}^{t_{mag}}$ indicates the difference between the function values in the square bracket when $z_{0}$ takes the values of $t_{mag}$ and zero.

Similarly, if the lower left corner of permanent magnet 2 is at position (0, *t_us_, p_mag_*), the magnetic field intensity generated by this magnet at point A(*x*, *y*, *z*) can be expressed by:

(6) $$B_{2x}=\int_{p_{mag}}^{p_{mag}+t_{mag}}dB_{2x}=-\frac{K}{2}[\Gamma \left(l_{mag}-x,y-t_{us},z\right)+\Gamma \left(l_{mag}-x,h_{mag}+t_{us}-y,z\right)-\;\Gamma \left(x,y-t_{us},z\right)-\Gamma \left(x,h_{mag}+t_{us}-y,z\right)]{|}_{p_{mag}}^{p_{mag}+t_{mag}},$$

(7) $$B_{2y}=\int_{p_{mag}}^{p_{mag}+t_{mag}}dB_{2y}=-\frac{K}{2}[\Gamma \left(h_{mag}+t_{us}-y,x,z\right)+\Gamma \left(h_{mag}+t_{us}-y,l_{mag}-x,z\right)-\;\;\Gamma \left(y-t_{us},x,z\right)-\Gamma \left(y-t_{us},l_{mag}-x,z\right)]{|}_{p_{mag}}^{p_{mag}+t_{mag}},$$

(8) $$B_{2z}=\int_{p_{mag}}^{p_{mag}+t_{mag}}dB_{2z}=-K[\varphi \left(y-t_{us},l_{mag}-x,z\right)+\varphi \left(h_{mag}+t_{us}-y,l_{mag}-x,z\right)+\;\varphi \left(x,h_{mag}+t_{us}-y,z\right)+\varphi \left(l_{mag}-x,h_{mag}+t_{us}-y,z\right)+\varphi \left(h_{mag}+t_{us}-y,x,z\right)+\;\varphi \left(y-t_{us},x,z\right)+\varphi \left(l_{mag}-x,y-t_{us},z\right)+\varphi \left(x,y-t_{us},z\right)]{|}_{p_{mag}}^{p_{mag}+t_{mag}}.$$

The total magnetic field at point A(*x*, *y*, *z*) represents the vector superposition of the magnetic fields generated by permanent magnets 1 and 2, which can be written as follows:

(9) $$B_{x}=B_{1x}+B_{2x},$$

(10) $$B_{y}=B_{1y}+B_{2y},$$

(11) $$B_{z}=B_{1z}+B_{2z}.$$

#### 3.2.2. Effect of Coil Parameters on Electromagnetic Force Torque

After obtaining the magnetic field distribution between two permanent magnets in a microshutter element, the electromagnetic force torque of a coil can be analyzed.

**Figure 7:**
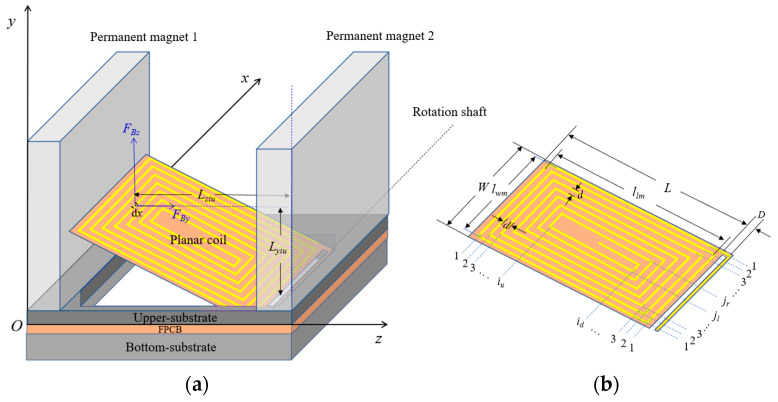
(**a**) The schematic diagram of a planar coil element leaning against the permanent magnet. (**b**) The structural parameters of the wires in a planar coil element.

As shown in Figure 7, for an infinitesimal element *dx* of a wire numbered as *i_u_* that is parallel to the *x*-axis in the coil, the electromagnetic force torque of the *z*-component of the magnetic field on the infinitesimal wire element can be expressed by:

(12) $$dM_{ziu}=IB_{z}\left(x,y,z\right)dx\cdot L_{ziu},$$

where *I* is the current in the coil and $L_{ziu}$ denotes the force arm of the electromagnetic force on the infinitesimal wire element, which can be expressed by:

(13) $$L_{ziu}=\left[\;\frac{L+D}{2}+\frac{l_{lm}}{2}-\left(i_{u}-1\right)d\right]cos\theta ,$$

where *L* is the length of the planar coil element; *D* represents the distance between the planar coil element’s edge to the torsion beam; *l_lm_* is the longest wire length along the coil’s length directions; *i_u_* is the wire number, and the numbering rule is shown in Figure 7b; *d* is the distance between two adjacent wires of the planar coil; *θ* is the angle between the coil plane and *xOz* plane.

If the coordinates along the *x*-axis of the two ends of the wire are denoted by *x*_1_ and *x*_2_, the electromagnetic force torque of the *z* component of the magnetic field on the wire can be expressed as follows:

(14) $$M_{ziu}=\int_{x_{1}}^{x_{2}}IB_{z}\left(x,y,z\right)dx\cdot L_{ziu}.$$

Similarly, the electromagnetic force torque of the *y* component of the magnetic field experienced by the wire can be expressed by:

(15) $$M_{yiu}=\int_{x_{1}}^{x_{2}}IB_{y}\left(x,y,z\right)dx\cdot L_{yiu}.$$

According to the force law of an electrified wire in the magnetic field, the electromagnetic force of the *x* component of the magnetic field on the wire is zero. Therefore, the torque of the electromagnetic force on the wire can be expressed as follows:

(16) $$M_{iu}=M_{ziu}+M_{yiu}.$$

Similarly, the torque of the electromagnetic force on each wire can be calculated, and the total torque of the electromagnetic force on the whole coil can be obtained by:

(17) $$M_{tol}=\sum_{i_{u}=1}^{n}M_{iu}+\sum_{i_{d}=1}^{n}M_{id}+\sum_{j_{l}=1}^{n}M_{jl}+\sum_{j_{r}=1}^{n}M_{jr},$$

where *n* is the number of turns of the plane coil; *i_d_*, *j_l_*, and *j_r_* denote the numbers of wires in the plane coil along different directions as shown in Figure 7b, and *M_id_*, *M_jl_*, and *M_jr_* are the corresponding torques of electromagnetic force on the wires, respectively.

It is worth noting that the above analysis holds for a single-layer planar coil but, for a double-layer planar coil, the total torque of the electromagnetic force on the planar coil will be doubled when compared to that of a single-layer planar coil.

Since the required values of the period of a microshutter array and clear aperture of a microshutter element are 4 mm and 2.8 mm, respectively, it is reasonable to set the permanent magnet thickness to 1 mm and the side length of the square hole in the bottom-substrate to 2.8 mm. Further, to cover a square hole, the coil plane element size should be larger than 2.8 mm. For making a coil plane element lean against the permanent magnet and reserving space on FPCB to extract electrodes, each coil plane element is designed to be rectangular with a length *L* = 3.6 mm, a width *W* = 3.1 mm, and a distance *D* = 0.15 mm. When a coil plane element is leaning against the permanent magnet, the angle between the coil plane and the *xOz* plane is approximately 37°.

**Figure 8:**
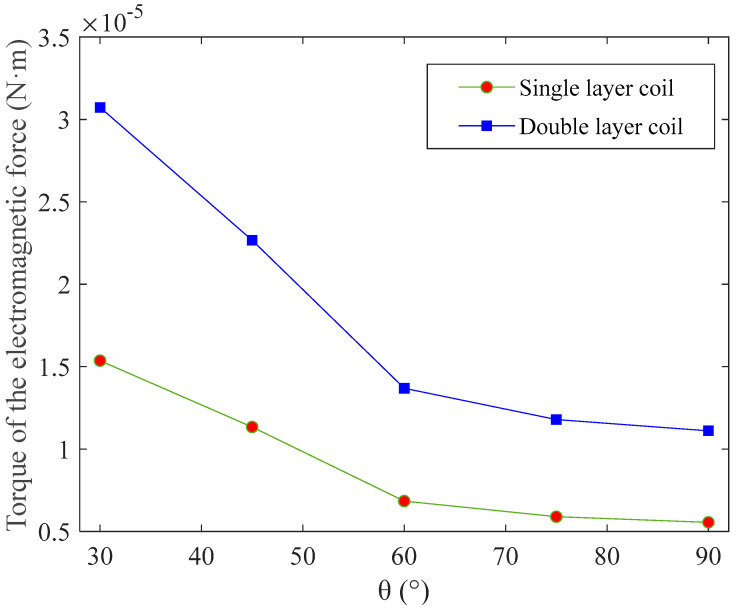
Electromagnetic force torque results for different angles between the coil plane and the horizontal plane.

Assuming that the structural and material parameters of permanent magnets 1 and 2 are identical, their length, height, thickness, and surface current density values are 4 mm, 3 mm, 1 mm, and 1 × 10^−6^ A/m [23], respectively. The thickness of the upper- substrate is 1 mm. The longest wire length values along the coil’s length and width directions are 3.3 mm and 2.8 mm, respectively. The linewidth of the coil wire is 0.1 mm, the adjacent wire distance is 0.15 mm, the number of turns of a single-layer coil is seven, and the current in the coil is 1 A. Using Equations (1)–(17), the total torque of the electromagnetic force on single- and double-layer coils are calculated for the angles between the coil plane and the *xOz* plane of 30°, 45°, 60°, 75°, and 90°, as shown in Figure 8.

As shown in Figure 8, the larger the angle between the coil plane and the *xOz* plane is, the smaller the total torque of electromagnetic force on a coil plane element will be. When the angle between the coil plane and the *xOz* plane is 90°, the total torque of the electromagnetic force on the single- and double-layer coils is the smallest and equals 5.18 × 10^−6^ N·m and 1.04 × 10^−5^ N·m, respectively. This result indicates that the resistance torque of the torsional beam must be less than this value to deflect the coil plane element.

#### 3.2.3. Effect of Torsion Beam Parameters on Resistance Torque

**Figure 9:**
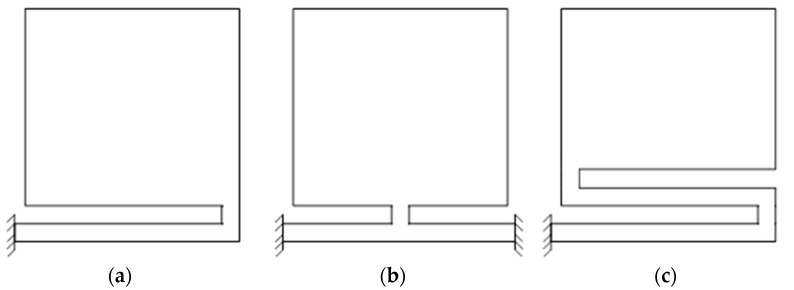
Microshutter elements with three typical torsion beam structures. (**a**) a single beam; (**b**) two beams connected in parallel; (**c**) two beams connected in series.

The resistance torque of a torsion beam is closely related to the structural parameters and size of the torsion beam, which has a strong influence on the deflection of a coil element. Microshutter elements with three typical torsion beam structures are presented in Figure 9. Figure 9a shows the torsion beam structure supported by a single beam; Figure 9b presents the torsion beam structure supported by two beams connected in parallel; Figure 9c displays the torsion beam structure supported by two beams connected in series.

For the torsion beam structure supported by a single beam, assume that the length, width, and thickness of the beam are denoted by *l_b_*, *w_b_*, and *t_b_* respectively; then, the torque on the torsion beam *T_b_* and its torsion angle *θ_b_* satisfy the following linear relationship:

(18) $$T_{b}=k\theta_{b},$$

where *k* is the elastic coefficient of the torsion beam, and it can be calculated by:

(19) $$k=\frac{GI_{p}}{l_{b}},$$

where *G* is the shear modulus of elasticity of the torsion beam material, and *I_p_* is the torsional moment of inertia of the torsion beam, and they are expressed as follows:

(20) $$G=\frac{E}{2\left(1+\mu \right)},$$

(21) $$I_{p}=\frac{w_{b}t_{b}^{3}}{16}\left[\frac{16}{3}-3.36\frac{t_{b}}{w_{b}}\left(1-\frac{t_{b}^{4}}{12w_{b}^{4}}\right)\right],$$

where *E* and *μ* are Young’s modulus and Poisson’s ratio of the beam material, respectively.

According to Equations (18)–(21), the longer the length, the thinner the thickness and the narrower the width of the torsion beam will be, and the easier it will be to twist. Because the width of the coil plane element is 3.1 mm, it is reasonable to set the length of the torsion beam to 3.2 mm. Considering the processing capacity of a FPCB, the thinnest fabrication thickness that can be realized is 0.1 mm, so the thickness of the torsion beam is set to 0.1 mm. In addition, since the coil electrodes need to be extracted through the torsion beam, the width of the torsion beam must not be less than the coil linewidth.

When two beams with elastic coefficients of *k*_1_ and *k*_2_ are connected in parallel or series, the total elastic coefficient is respectively calculated by:

(22) $$k_{p}=k_{1}+k_{2},$$

(23) $$k_{s}=\frac{k_{1}k_{2}}{k_{1}+k_{2}}.$$

**Figure 10:**
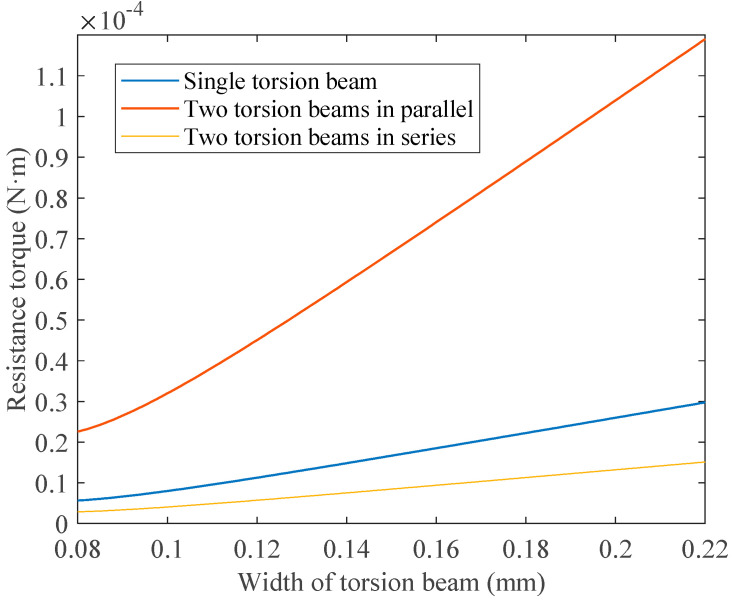
Resistance torque results of the three structures with different beam widths when the planar coil element is deflected to 90°.

As the length of a single beam presented in Figure 9a is 3.2 mm, when two beams are connected in parallel (the structure in Figure 9b) the length of each beam is 1.6 mm; but when two beams are connected in series, the structure in Figure 9c, the length of the second beam is slightly shorter than that of the first beam. In this study, the length of the second beam is set to 3.1 mm. Considering that the material of a FPCB is mainly polyimide with Young’s modulus of 3.1 GPa and Poisson’s ratio of 0.34, then according to Equations (18)–(23) and the aforementioned torsion beam parameters, the resistance torque for the three structures with different beam widths when the planar coil element is deflected to 90° can be calculated, as shown in Figure 10.

As shown in Figure 10, the resistance torque of the structure with two torsion beams connected in parallel is the largest among all structures. Although the width of the torsion beam is less than 0.1 mm, the corresponding resistance torque is still much larger than the electromagnetic force torque calculated in Section 3.2.2, so this structure is not applicable. When the single torsion beam structure with a beam width of less than 0.11 mm or the structure with two torsion beams connected in series with a beam width of less than 0.17 mm is used, the resistance torque will be less than the electromagnetic force torque calculated in Section 3.2.2, which is suitable for the microshutter element design.

#### 3.2.4. Effect of Coil Element Parameters on Switching Response Time

The response time is an important parameter of a microshutter, which affects the dynamic detection capability of a FOV-gated optical imaging system. For a microshutter element, the response time is affected not only by the elastic coefficient but also by the torque of inertia and quality factor of the microshutter element. The response time of a microshutter element can be calculated by [24]:

(24) $$T=\frac{\pi -\alpha }{\sqrt{\frac{k}{J}\left(1-\frac{1}{4Q^{2}}\right)}},$$

where *Q* is the quality factor, which reflects the air damping, and the smaller the value of *Q* is, the greater the air damping is; the value of *Q* is generally set to 10 on the micron or millimeter scale; *α* is the damping angle, and its value satisfies the conditions of $cos\alpha =\frac{1}{2Q}\;$; *J* is the mass moment of inertia of inertia, and it is expressed by:

(25) $$J=\frac{1}{3}\rho WtL\left(\frac{t^{2}}{4}+L^{2}+3LD+3D^{2}\right),$$

where *ρ* is the material density of the torsion beam; *W* is the width of the planar coil element; *t* is the thickness of the planar coil element; *L* is the length of the planar coil element, and *D* represents the distance from the microshutter’s edge to the torsion beam, as shown in Figure 7a.

**Figure 11:**
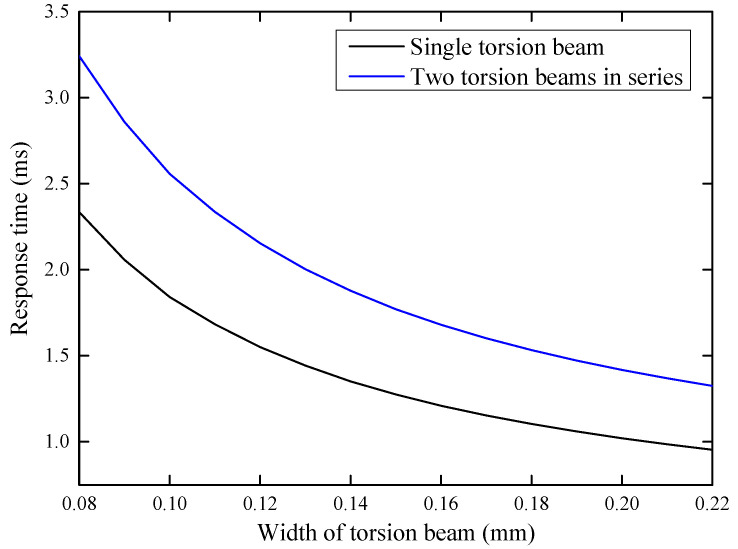
The response time results of the single torsion beam and the structure with two torsion beams connected in series at different beam widths.

Considering that the material of the torsion beam is mainly polyimide with material density of 1.4 kg/m^3^ and the thickness of the planar coil element is 0.1 mm, using Equations (24) and (25), the response times of the single torsion beam and the beam structure with two torsion beams connected in series at different beam widths are calculated, as shown in Figure 11.

As presented in Figure 11, the response time of the single torsion beam is shorter than that of the beam structure with two torsion beams connected in series, so the single torsion beam is more conducive to the rapid switching of a microshutter array. Moreover, using the beam structure with two torsion beams connected in series will provide a lower duty cycle. Thus, it is desirable to use a single torsion beam to support a planar coil element.

Based on the above analysis results, when a planar coil is designed to be double-layered, the wire width of the coil is 0.1 mm, the number of turns of the single-layer coil is seven, and a single torsion beam with the 0.1-mm thickness, 3.2-mm length, and 0.1-width is adopted, the switching function of the planar coil can be realized and the response time of a microshutter element can reach a value of 1.8 ms. The main parameters of the designed microshutter element are shown in Table 2.

## 4. Experimental Results

**Figure 12:**
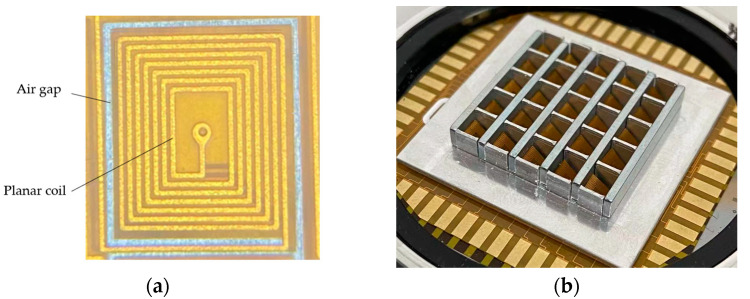
(**a**) The microscopic picture of a fabricated planar coil element. (**b**) Fabricated microshutter array sample based on the electromagnetic actuation.

To verify the feasibility of the design described above, a microshutter array sample with 5 × 5 elements was fabricated and tested. In the experiment, a 0.1-mm thick FPCB was used to process a double-layered planar coil array. The torsion beam was a single-beam structure. The size of the circuit board was 4 mm × 4 mm; the thickness of polyimide in the material was 12.5 μm; the copper foil material was electrolytic copper with glue, and the thickness of the outer copper was 12 μm. The main parameters of the fabricated microshutter element are consistent with that in Table 2, and the microscopic picture of a fabricated planar coil element is shown in Figure 12a. The coil element size was 3.6 mm × 3.1 mm, the linewidth of the wire in the coil was 0.1 mm, and the adjacent wire distance was 0.15 mm. The torsion beam adopted the single torsion beam structure, with a width and length of 0.1 mm and 3.2 mm, respectively.

Next, the FPCB with the planar coil array was bonded to a bottom-substrate, while the planar coil array corresponding to the square hole array fabricated on the bottom-substrate one by one. The thickness of the bottom-substrate was 0.2 mm, and the side length of the square hole was 2.8 mm. A lateral light shield array was fabricated on an upper-substrate with 1 mm thickness. The NdFeB permanent magnet array and the lateral light shield array were integrated with the planar coil array so that each planar coil element leaned against the permanent magnet. Finally, the microshutter and microlens arrays were integrated to obtain the microshutter and microlens array integrated device, as shown in Figure 12b.

The NdFeB permanent magnet had a thickness of 1 mm, a height of 3 mm and a length of 20 mm. A permanent magnet with a length of 20 mm was shared by five microshutter elements, so a total of six permanent magnets were used in 5 × 5 microshutter elements.

**Figure 13:**
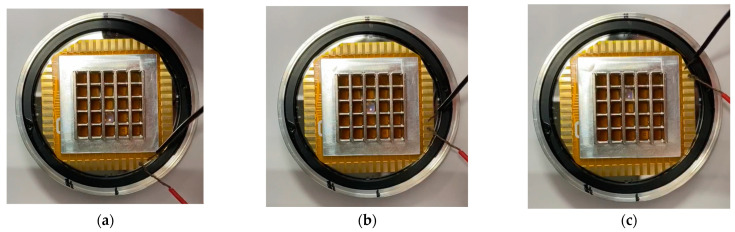
An experiment to verify the switch function of the fabricated microshutter array. (**a**–**c**) show respectively the opened state of different microshutter elements when a 3-V voltage was applied to the corresponding coil element.

A digital multimeter (UNI-T UT603, Uni-Trend technology Co., Ltd., Dongguan, China) was used to measure the resistance of a planar coil element. The measured resistance of the coil element was approximately 3 Ω. A voltage of 3 V was applied to the coil element by a DC power source (Keysight E3632A, Keysight Technologies, Santa Rosa, CA, USA), and the current in the coil was roughly 1 A. We found that the coil element could be completely opened, as shown in Figure 13a. After the voltage was removed, the microshutter element returned to the closed state under the influence of the restoring force of the torsion beam. When a 3-V voltage was applied to the other coil element, the corresponding microshutter element was also turned on, as shown in Figure 13b,c. The experimental results were consistent with the theoretical analysis results.

**Figure 14:**
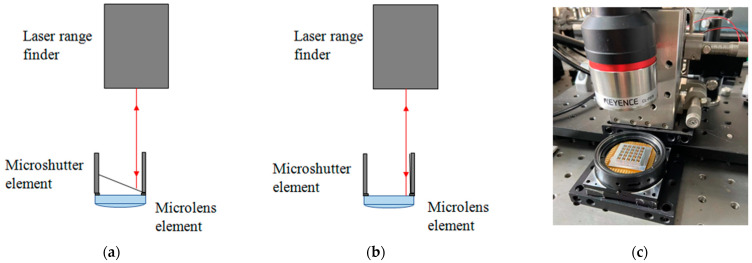
Schematic diagram and experimental setup of the response time measurement of a microshutter element by a laser range finder. (**a**) shows the laser was incident on the surface of the microshutter element before applying voltage to the planar coil element; (**b**) shows was the laser incident on the back surface of the microlens element after applying voltage to the planar coil element. (**c**) shows the experimental setup for measuring the response time of a microshutter element.

Next, the response time of a microshutter element was tested by a laser range finder. First, the laser of the laser range finder was visible on the surface of a microshutter element. The position of the laser spot on the surface of the microshutter element was close to the torsion beam, as shown in Figure 14a. Then, a 3-V voltage was applied to the microshutter element, and the microshutter element was deflected; the laser was visible on the back surface of the microlens element, as shown in Figure 14b. The time required to switch from state (a) to state (b) in Figure 14 was considered as an opening time of the microshutter element. The closing time of the microshutter element after the voltage was removed was measured using the same method.

**Figure 15:**
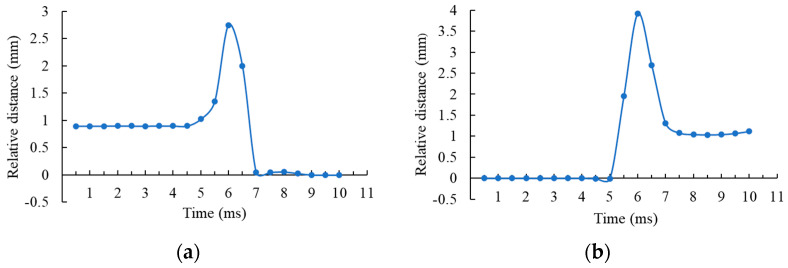
Test results of the response time of a fabricated microshutter element. (**a**) shows the opening process of the microshutter element after applying a 3-V voltage; (**b**) shows the closing process of the microshutter element after removing a 3-V voltage.

The picture of the experimental setup used to measure the response time of a microshutter element is shown in Figure 15c. The laser range finder used in the experiment was a KEYENCE CL-P070 (Keyence Corporations, Osaka, Japan). The sampling time interval of the laser range finder was 0.5 ms, which meets the requirement of the time resolution for measuring the response time of a microshutter element.

In the experiment, the back surface of the microlens element was taken as the zero-displacement plane, and the relative displacement of the laser spot on the microshutter element’s surface relative to the zero-displacement plane was defined as positive displacement when the position of the laser spot on the microshutter element’s surface was higher than the zero-displacement plane. Figure 15 shows the variation of the relative displacement with time. It can be seen from Figure 15a that the relative displacement increased first and then decreased rapidly in the process of switching from state (a) to state (b) in Figure 14 after the voltage was applied. This was because the distance between the laser spot on the microshutter element’s surface with the zero-displacement plane gradually increased during the deflection of the microshutter element. However, when the deflection angle of the microshutter element was close to 90°, the laser left the microshutter element and was visible on the back surface of the microlens element. Therefore, the relative displacement rapidly changed to about 0. The time difference from the beginning of deflection of the microshutter element to the laser incident on the back surface of the microlens element was the opening time of the microshutter element. It can be seen from the Figure 15a that the opening time of the microshutter element was approximately 2.5 ms. Similarly, the closing time required to recover from state (b) to state (a) in Figure 14, after the voltage was removed, was also roughly 2.5 ms, as shown in Figure 15b. The test results were basically consistent with the theoretical analysis results, which proved the feasibility of using a microshutter array based on the electromagnetic actuation in a FOV gated imaging system.

## 5. Conclusions

Aiming at the application requirements of a FOV-gated imaging system for all-time star detection, this paper proposes a microshutter array with a large element size, high duty cycle, and high response speed based on electromagnetic actuation. The concept of the electromagnetically-driven microshutter array is introduced and the mathematical model of the electromagnetic torque of the planar coil in the magnetic field is constructed. The effects of the structural parameters of a coil element on the electromagnetic torque are analyzed, the influence of the three typical torsion beam structures on the resistance torque is discussed, and the conditions for the coil plane element to realize the switching function are defined. The response time of the proposed microshutter is calculated and the structure parameters of the coil element and torsion beam are determined. A sample of the proposed electromagnetically-driven microshutter array with a 4-mm period and a 0.7-duty cycle is fabricated. The optical switch function of the planar coil element is verified experimentally, and the response time of the microshutter element is tested. The test results show that the response time of the microshutter element is approximately 2.5 ms, which is basically consistent with the theoretical analysis results and meets the application requirements of the FOV-gated imaging system for all-time star sensors. This study avoids the problem of the stress control in bulk silicon micromachining and adopts the flexible circuit board processing technology with easy processing and a low cost for fabricating the large-size microshutter element, which can promote the practical application of the FOV-gated imaging system. Though the switching function of the proposed microshutter array was verified, there is a problem with high power consumption due to the high loading current in the planar coil. Therefore, in the following research work, the structure parameters of a microshutter element should be further optimized to reduce power consumption. Additionally, the number of elements, the duty cycle, and the response time of a microshutter element should be improved to meet more application requirements.

## Figures and Tables

**Table 1:** Requirements for the main design indexes of a microshutter element.

Parameter	Design Specification
Microshutter element size	4 mm × 4 mm
Clear aperture of a microshutter element	2.8 mm × 2.8 mm
Response time	≤10 ms

**Table 2:** The main parameters of the designed microshutter element.

Parameter	Value
Size of microshutter element	4 mm × 4 mm
Size of coil element	3.6 mm × 3.1 mm
Longest wire length along coil’s length directions	3.3 mm
Longest wire length along coil’s width directions	2.8 mm
Linewidth of wire	0.1 mm
Adjacent wire distance	0.15 mm
Number of coils turns	7 turns in a single layer (14 turns in a double layer)
Torsion beam structure	Single torsion beam
Torsional beam length	3.2 mm
Torsion beam width	0.1 mm
Torsion beam thickness	0.1 mm
Distance between coil element’ edge with torsion beam	0.15 mm

## Data Availability

The data presented in this study are available from the corresponding author upon reasonable request.
